# Seroprevalence and incidence of primary dengue infections among children in a rural region of Maharashtra, Western India

**DOI:** 10.1186/s12879-019-3937-z

**Published:** 2019-04-02

**Authors:** Paresh S. Shah, Kalichamy Alagarasu, Shivaji Karad, Avinash Deoshatwar, Santosh M. Jadhav, Tushar Raut, Anand Singh, Cecilia Dayaraj, Vasant S. Padbidri

**Affiliations:** 1 Dengue/Chikungunya Group, ICMR- National Institute of Virology, Pune, 411001 India; 2 Bioinformatics Group, ICMR- National Institute of Virology, Pune, 411001 India; 3 Epidemiology Group, ICMR- National Institute of Virology, Pune, 411001 India; 4 KEM Hospital and Research Centre, Pune, 411001 India

**Keywords:** Dengue, Seroprevalence, Incidence, IgG, Disease burden, Urbanization

## Abstract

**Background:**

Dengue infections have become a huge threat to public health systems in developing countries. Data on seroprevalence and incidence of dengue infections are lacking from rural regions of India. The objective of present study was to investigate the seroprevalence and incidence of dengue infection utilizing repeated serosurveys from a rural region of Maharashtra, Western India.

**Methods:**

In the present study, 819 children between ages 5 to 15 years from 21 villages in Pune District of Maharashtra, India were sampled in 2014 and 2016. The sera were tested for the presence of dengue specific IgG using an indirect IgG ELISA kit.

**Results:**

Overall seroprevalence of dengue was 15.3% (95% confidence intervals (CI) 12.9–17.8%) in 2014 and 20.5% (95% CI 17.8–23.4%) in 2016. Among the 694 children who were seronegative at baseline (2014), 78 seroconverted. Overall incidence rate of primary dengue was 54.2 infections/1000 children years (95% CI 43.0–67.3). Incidence of primary dengue infection was higher in children from urbanized villages compared to rural villages (Incidence rate ratio (IRR) 2.6 (95% CI 1.3–5.2)). In rural villages, incidence of primary dengue infection was higher in children aged 10 years or above as compared to those aged below 10 years (IRR 9.75 (95% CI 1.21–77.9).

**Conclusions:**

The study provides the incidence rates of primary dengue infections from a rural region of India. More multi centric studies investigating the incidence of dengue will provide accurate estimate of incidence of dengue and help formulate well directed policies. The results also suggest that urbanization and transitions in demographic settings might favour dengue outbreaks in rural regions and these regions need to be targeted for vector control measures.

## Background

Dengue, a mosquito borne viral disease, has emerged as a significant threat and burden to public health systems in tropical and sub tropical countries. The disease is caused by four serotypes of the dengue virus (DENV) and the outcome of the infection ranges from sub clinical infection, mild dengue to severe forms of dengue which occasionally are life-threatening if not clinically managed properly. Incidence of dengue has increased dramatically over the past five decades with 50–100 million infections occurring annually. Compared to the global burden of disease (GBD) 2013 estimates, GBD 2015 reported an increase in the number of dengue deaths by 48.7% resulting in 18,400 deaths [1]. The 2016 Yellow Book dengue maps have recategroized many geographical areas with sporadic/uncertain risk as regions with frequent/continuous risk while three states in Northern India which were previously uncategorized were determined to be regions with sporadic/uncertain risk [2].

The National Vector Borne Disease Control Program, Government of India has reported more than 100,000 cases during 2015–2017 [3]. However, a study estimated 390 million cases annually in India, while another study estimated annual average of 5,77,8406 cases suggesting severe under reporting [4, 5]. The surveillance system in India for reporting dengue cases includes 609 sentinel surveillance laboratories and 14 apex referral laboratories. Recently viral diagnostic laboratories have been added to the list, which reports the dengue cases [6]. Though, the surveillance system is being strengthened for reporting dengue, there is a lack of proper sero-epidemiological studies for estimating the dengue disease burden in India.

In India, only a few studies have investigated the seroprevalence of dengue and most of them have been conducted only in metropolitan cities [7–11]. To date, there is lack of studies reporting the incidence of dengue in different demographic settings in India. Such studies are essential to estimate the true burden of dengue and allocate resources accordingly for dengue surveillance and control. We carried out a study on 819 children aged 5 to 15 years belonging to 21 villages with different demographic settings in Vadu Region of Pune District, Western India to find out the seroprevalence and incidence of dengue in this cohort.

## Methods

### Study settings and population

Vadu is ~ 35 km north-east of Pune which is endemic for dengue. Vadu Rural Health Program (VRHP) runs a health and demographic surveillance system and has been involved in various field-based epidemiological studies and field trials of vaccines for more than a decade. VRHP has 22 villages and population of more than 1,00,000 that varies greatly over the years due to industrial zones in the area. VRHP has kept the population under close demographic surveillance since 2003. VRHP demographic surveillance system staff visits every household every six months and collects information on any changes in demographic characteristics of the household.

The villages were characterized as rural or urbanized according to the Indian Government Census (2011) criteria, which states that a place is defined as urban if it satisfies the following three criteria: (i) a minimum population of 5000; (ii) at least 75% of the male working population engaged in non-agricultural pursuits; and (iii) a population density of at least 400/km^2^ (1000/mile^2^). Since none of villages fulfilled the criteria, villages with minimum two criteria were considered as urban while others were considered as rural. Six villages were considered as urbanized villages while the remaining 15 villages were considered as rural.

### Sample size and study subjects

A WHO guidebook on sample size determination in health studies, a practical manual, was followed for sample size determination [12]. Based on the pilot study results [7] and considering 10% drop-outs [for loss to follow up for various reasons] every year, the sample size was calculated to be 1000. Our pilot study reported that there was an one-step increase in the seroprevalence of dengue from children aged below 10 years (17.2%) to children aged above 10 years (72.2%) in an urbanized village. Moreover, the study revealed that the seroprevalence was less than 10% in the children aged below five years. Considering that the exposure to dengue occurs in majority of the subjects aged above 10 years, 5–15 years age group of children was selected for the present study [8]. Vadu DSS database was used as a sampling frame for random selection of children between 5 and 15 years of age. Only subjects who were likely to stay in the area for the full study period were only included. The total children sampled from each village were proportional to the children population within 5–15 years of age. Using the sampling frame, children to be sampled were determined by random selection and their parents/legal guardian were contacted by the research staff. A written informed consent was obtained from the parents/legal guardian of the participating children. The study was approved by the institutional human ethics committees of the ICMR-National Institute of Virology, Pune, and KEM Hospital and Research Centre, Pune. Blood sample was collected by finger prick method. Using a sterile lancet, a left hand finger was pricked and 200 μl of blood was drawn using a sterile tip and a micropipette and collected in a 1.5 ml micro-centrifuge tube containing citrate. The samples were transported to ICMR-NIV maintaining cold chain. On arrival, samples were stored at -20 °C till testing. A total number of 823 children were sampled sequentially in 2014 and in 2016.

### In-direct IgG ELISA

To test whether the subjects had been exposed to dengue previously, the plasma was separated and tested for the presence of dengue virus-specific IgG using an in-direct IgG ELISA kit (EDEN01G, Panbio Inc., Brisbane, Queensland, Australia) according to the instructions of the manufacturer.

### Statistical analysis

Simple proportional analyses with confidence intervals for community prevalence were calculated using Epi-info version 7. Associations of demographic characteristics and dengue seroprevalence were assessed by univariate analysis using the Chi-square test. Incidence of dengue infection was calculated using incidence rate ratio (IRR) calculator available in STATA statistical software release 14.

## Results

### Seroprevalence of dengue among the children in 2014 and 2016

Among the 823 children, four had equivocal results for 2016 and were excluded for further analysis resulting in a sample size of 819 children. The overall seroprevalence of dengue was 15.3% (95% confidence intervals (CI) 12.9–17.8%) in 2014 and 20.5% (95% CI 17.8–23.4%) in 2016.

### Association of demographic characteristics with dengue seroprevalence

When the children were grouped into those with age below 10 years old and those with age of ten years or greater, the seroprevalence was significantly higher in children with age of 10 years old or greater for both sero-surveys. The seroprevalence was not different between male and females (Table 1).

**Table 1:** Demographic parameters and prevalence

Variable	Sero-prevalence			
	First visit in 2014		Second visit in 2016	
	N (%)	Prevalence Ratio with 95% CI	N (%)	Prevalence Ratio with 95% CI
Age group (Year)^a^				
Below 10 (*n* = 427)	49 (11.5)	–	68 (15.9)	–
10 years or above (*n* = 392)	76 (19.4)	**1.69 (1.21, 2.35)** ^b^	100 (25.5)	**1.60 (1.22, 2.11)** ^c^
Gender				
Female (*n* = 404)	71 (17.6)	–	91 (22.5)	–
Male (*n* = 415)	54 (13.0)	0.74 (0.53, 1.03)	77 (18.6)	0.82 (0.63, 1.08)
Demographic setting				
Rural (*n* = 215)	25 (11.6)	–	28 (13.0)	–
Urban (*n* = 604)	100 (16.6)	1.42 (0.95, 2.15)	140 (23.2)	**1.78 (1.22, 2.59)** ^d^

^a^Age group is based on age at year of entry (2014) into the study; ^b^*P* = 0.0017; ^c^*P* = 0.0007; ^d^*P* = 0.0015

### Association of demographic settings with dengue seroprevalence

The association between the demographic status/settings of the villages and dengue seroprevalence was investigated and the results revealed that urbanized villages had higher seroprevalence compared to rural villages and and the difference was significant in 2016 (Table 1).

### Changes in the status of antibodies in paired sera collected during 2014 and 2016 and incidence of dengue infections

Among the 819 children, 75.2% remained seronegative during both 2014 and 2016 while 9.5% converted to seropositive from seronegative status in 2016. Ninety children (11.0%) remained seropositive during both the years while 4.3% of the children became seronegative from seropositive status (Table 2).

**Table 2:** Changes in the status of antibody to dengue virus in paired sera collected in children from 2014 and 2016

Presence of antibody to dengue virus in 2014	Presence of antibody to dengue virus in 2016	Number of subjects	Percentage with 95% confidence intervals
Negative	Negative	616	75.2 (72.1–78.0)
Negative	Positive	78	9.5 (7.7–11.7)
Positive	Positive	90	11.0 (9.0–13.3)
Positive	Negative	35	4.3 (3.1–5.9)

The children who were negative during 2014 were considered for calculating the incidence of primary dengue infection. There were 78 new infections among the 694 seronegative children and the overall incidence rate of primary dengue was 54.2 /1000 children years (95% CI 43.0–67.3).

### Influence of demographic settings and demographic characteristics on the incidence of primary dengue infections

The incidence of primary dengue infections was significantly higher in urbanized villages compared to rural villages (IRR 2.6 (95% CI 1.3–5.2)) (Table 3).

**Table 3:** Estimated incidence of primary dengue infections

	Incidence rate (IR) (per 1000 child years)	Incidence rate ratio (95% Confidence Limits)
Gender		
Female	54.9	-
Male	53.3	0.97 (0.62–1.51)
Demographic setting		
Rural	24.6	-
Urban	64.2	**2.6 (1.3–5.2)** ^#^
Demographic setting and Age		
Rural		
Below 10	5.0	-
Above 10	48.6	9.75 (1.21–77.9)*
Urban		
Below 10	56.4	-
Above 10	73.6	1.30 (0.81–2.09)

^#^*P* = 0.0050; ^*^P = 0.0083

Gender had no influence on the incidence of primary dengue (IRR with 95% CI 0.97 (0.62–1.51)). A significantly higher incidence of primary dengue was observed among children aged 10 years and above as compared to the lower age group from rural villages (IRR 9.75 (95% CI 1.21–77.9)). In urbanized villages, no significant difference in incidence was observed between the age groups (Table 3).

## Discussion

Different studies based on passive surveillance and modeling data have predicted a higher incidence of dengue in India as compared to other countries. There are only few studies which have reported the seroprevalence of dengue in different groups of the general population (Table 4) [7–11]. In the present study, the seroprevalence and incidence of primary dengue among children aged 5 to 15 years from a rural region of Pune district, Maharashtra, India, was investigated. The results revealed an increase in seroprevalence over the two serosurveys, but, it was lower as compared to seroprevalence reported for children in metropolitan cities in India and highly endemic countries. Our earlier pilot study in two villages also reported difference in the seroprevalence between rural and urbanized villages [8]. In the present study, demographic characteristics like age group [< 10 years vs > = 10 years] and settings [rural vs urbanized village] were associated with higher seroprevalence in the year 2016. This suggests that dengue seroprevalence might vary between places based on the level of urbanization.

**Table 4:** Data on seroprevalence of dengue in India based on different studies

Year of Study	Place of Study	Category of population studied	Age group	Number of subjects tested	Seroprevalence percentage	Reference
2011–2012	New Delhi	Children	5–10 year	649	63.3	7
	Kalyani, West Bengal			323	23.2	
	Wardha, Andhrapradesh			323	69	
	Mumbai, Maharashtra			301	80.1	
	Hyderabad, Telungana			639	58.4	
	Bengaluru, Karnataka			323	62.5	
2011	Chennai, Tamil Nadu	General population	5-40 year	800	93	9
2011	Pimple Jagtap, Pune, Maharashtra	General population	All age groups	702	42.8	8
	Koregaon Bhima, Pune, Maharashtra			153	58.8	
2012	New Delhi	Blood donors	19-51 year	200	58.8	11
2012	New Delhi	Household contacts and neighborhood contacts	All age groups	2125	34.2	10

The indirect IgG-ELISA for dengue, used in this study, is reported to have high sensitivity and specificity and has been utilized in dengue serosurveys [7, 9, 13]. However, extensive cross-reactivity is known to occur among Flaviviruses especially in the context of IgG reactivity [14]. Three other Flaviviruses, other than DENV, circulate in India; Japanese encephalitis virus, West Nile virus and Kyasanur Forest Disease virus [15–17]. However, none of these viruses have been reported in the study region and therefore the seropositivity observed was in all probability against DENV.

Overall incidence of primary dengue infection in children aged 5–15 years was 54/1000 person years. The incidence also varies according to the demographic settings with urbanized villages having a higher incidence as compared to rural villages. The overall incidence reported in the present study is much lower than that reported in other studies. A study from a Brazilian birth cohort in the city of Recife which followed the children for first two years of life has reported an incidence of 107.6/1000 person years [18]. The Nicaraguan pediatric cohort study conducted in the city of Managua has reported an overall incidence of 90.2 infections/1000 person years. The incidence rates of primary and secondary dengue infections were 78.8 and 99.5/1000 person years respectively [19]. A cohort study from an urban community in Cebu city, Philippines reported incidence rates of 121.9 and 149.4/1000 person years among children groups aged between 6 months and 5 years and between 6 and 15 years respectively [20]. Among Colombian children living in the city of Medellin, age-specific incidence rates ranged from 57.6/1000 person years in children aged 1–5 years old to 90/1000 person years in children aged between 11 and 15 years [21]. A study on children from urban areas of Indonesia estimated that 13.1% of children experience primary infection each year [22]. In the present study, the age specific incidence rates of primary dengue were 43.3 and 67.3/1000 person years in children below 10 years old and children aged 10 or above respectively and it was significantly higher in urbanized villages compared to rural villages. Although, the incidence rates of primary dengue reported in the present study are lower compared to other studies performed in urban settings, it is possible that incidence rates of primary dengue in Indian metropolitan cities may be higher or comparable to those reported in countries hyperendemic for dengue. A study on Israeli expatriates living in Delhi, a metropolitan city in India, conducted between August and September 2015 revealed that the attack rates of dengue in children (4.7%) and adults (6.3%) were similar and hypothesized that the incidence of dengue was highly underestimated [23]. A study done in Malaysia comparing urban and rural areas have reported that the seroprevalence of dengue in urban areas have become stabilized, while in rural areas it was steadily increasing [24]. Urban regions are characterized by a dense population, more indoor artificial containers, poor vegetation, and a high degree of shade by surrounding buildings compared to rural regions and such conditions favour *Aedes aegyptii* [25]. A study from Singapore reported that the population growth contributed 86% of the dengue incidence compared to 14% contribution by increase in temperature [26]. The steady increase in population and changes occurring in demographic settings might favour dengue outbreaks in rural regions which are in the process of urbanization, and these regions need to be targeted for vector control measures. Urbanization, globalization, and international mobility which together are also called as modern world triad increases the threat posed by *Aedes* transmitted viral diseases such as dengue, chikungunya, zika, and yellow fever. The modern world triad has led to the unprecedented emergence of these epidemic aroboviral diseases in the past five decades and underscores the need for better public health infrastructure [27, 28].

In the present study, waning of antibody response was observed in 4.3% of children. A similar phenomenon has been observed in studies conducted in Taiwan and China [29, 30]. The study from China reported that the IgG prevalence was higher in symptomatic subjects compared to asymptomatic individuals [30]. It is also possible that the subjects with antibody response might be frequently exposed to the virus leading to a sustained response while those with waning of antibody response may not have encountered the virus after their initial exposure. These children might be having antibody levels that are not detectable by the ELISA kit used. A recent study of a Nicaraguan pediatric cohort has shown that the risk of severe dengue disease is highest within a narrow range of pre existing antibody titre [31]. Hence it is possible that these children in whom antibody waning was observed might have a higher risk of developing severe disease if infected for the second time. However, more longitudinal cohort studies are needed to understand the host and environmental (repeated exposure to the virus) factors affecting waning of antibody response.

The study has some limitations. Though the children were sampled at two time points, no details of the symptoms developed by them were available. Hence, it was not possible to find out the ratio of symptomatic to asymptomatic infections. Since blood samples were collected by finger prick, sufficient volume of sample was not available to perform plaque reduction neutralization tests to find out the serotypes to which the children were exposed. The study also lacks data on vector population in the region.

## Conclusions

This study estimates the incidence of primary dengue infections among children from a rural region of India. The incidence varies according to the demographic settings with urbanization having a major impact. More multi centric studies in all age groups involving rural, urban and metropolitan regions are needed from India which will help in providing accurate estimates of new dengue infections and actual disease burden. Such studies will also help in allocating appropriate resources to control vector borne diseases.

## Abbreviations

CI: Confidence intervals
DENV: Dengue virus
DSS: Demographic surveillance system
GBD: Global burden of disease
ICMR: Indian Council of Medical Research
IRR: Incidence rate ratio
VRHP: Vadu Rural Health Program
WHO: World Health Organization
